# Readout From Iconic Memory and Selective Spatial Attention Involve Similar Neural Processes

**DOI:** 10.1111/j.1467-9280.2007.01998.x

**Published:** 2007-10

**Authors:** Christian C Ruff, Árni Kristjánsson, Jon Driver

**Affiliations:** 1 Institute of Cognitive Neuroscience and Department of Psychology, University College LondonLondon, United Kingdom; 2 Wellcome Department of Imaging Neuroscience, University College LondonLondon, United Kingdom; 3 Department of Psychology, University of IcelandReykjavik, Iceland

## Abstract

Iconic memory and spatial attention are often considered separately, but they may have functional similarities. Here we provide functional magnetic resonance imaging evidence for some common underlying neural effects. Subjects judged three visual stimuli in one hemifield of a bilateral array comprising six stimuli. The relevant hemifield for partial report was indicated by an auditory cue, administered either before the visual array (precue, spatial attention) or shortly after the array (postcue, iconic memory). Pre- and postcues led to similar activity modulations in lateral occipital cortex contralateral to the cued side. This finding indicates that readout from iconic memory can have some neural effects similar to those of spatial attention. We also found common bilateral activation of a fronto-parietal network for postcue and precue trials. These neuroimaging data suggest that some common neural mechanisms underlie selective spatial attention and readout from iconic memory. Some differences were also found; compared with precues, postcues led to higher activity in the right middle frontal gyrus.

Iconic memory (for reviews, see Long, 1980; Neisser, 1967; Sperling, 1967) and selective attention (for reviews, see Driver, 2001; Pashler, 1998) are two classic topics in cognitive psychology that have traditionally been considered separately. From a functional perspective, however, the paradigms commonly used to study these constructs are often quite similar (see Bundesen, 1990; Gegenfurtner & Sperling, 1993; Shih & Sperling, 2002). Paradigms in both areas have often employed cued partial-report tasks involving multicharacter displays, to reveal some form of visual capacity limitations. In the study reported here, we employed functional magnetic resonance imaging (fMRI) to show that two variants of such a paradigm, traditionally considered to index selective attention and iconic memory, may recruit common neuronal mechanisms.

Selective attention is often characterized as reflecting on-line allocation of limited processing resources (e.g., Broadbent, 1958; Bundesen, 1990; Driver, 2001; Pashler, 1998) and has often been studied by precuing attention to a particular location prior to onset of the stimuli to be judged. Many behavioral studies have shown that such precuing of spatial attention can facilitate sensory processing at the cued location (e.g., Driver, 2001; Kristjánsson & Nakayama, 2003; Pashler, 1998; Posner, Snyder, & Davidson, 1980). Moreover, neuroimaging studies have revealed that activity in occipital visual cortex can be enhanced by such precued spatial attention (e.g., Driver & Frackowiak, 2001; Serences & Yantis, 2006), possibly as a result of top-down modulations from a fronto-parietal attentional network (e.g., Desimone & Duncan, 1995; Kastner & Ungerleider, 2000; Ruff et al., 2006).

Although the classic literature on iconic memory is largely separate from that on attention, there are some conceptual similarities. Sperling (1960) first observed that although observers can typically report only a total of three or four characters from brief multicharacter displays (an indication of some form of limited processing capacity), they can often correctly report each of the characters in one specific cued subset (e.g., any given column of a 4 × 4 grid) if the cue for partial report follows shortly after the display—a form of rapid postcuing. This effect was classically taken as evidence that visual information might first enter a relatively high-capacity but short-duration iconic memory, from which information might be read out via a postcue, if this cue is given before the iconic representation decays (Neisser, 1967; Sperling, 1960). Although the nature, locus, and possible utility of iconic memory have been extensively debated (see, e.g., Coltheart, 1980; Di Lollo, 1977; Di Lollo & Bischof, 1995; Haber, 1983; Irwin & Yeomans, 1986; Long, 1980; Sakitt, 1976), the basic behavioral phenomena are well established.

Research on iconic memory bears conceptual relations to the largely separate research on selective attention, as some theoretical analyses have already emphasized (e.g., Bundesen, 1990). In both cases, limited processing capacity is thought to be allocated via cues, administered either before (in prototypical attention studies) or rapidly following (in iconic-memory studies) a display. Recent behavioral studies (e.g., Becker, Pashler, & Anstis, 2000; Lamme, 2003; Landman, Spekreijse, & Lamme, 2003) and some philosophical work (Block, in press) have led to renewed interest in the mechanisms underlying effective postcues. There remains a logical difference between precuing, which can modulate the system prior to presentation of critical stimuli, and postcuing, which can modulate the system only after critical stimuli have been presented. However, this theoretical distinction may be somewhat artificial as a strict dichotomy, given that visual processing can continue after stimulus offset and is now known to be highly recursive (e.g., Bullier, 2001; Lamme & Roelfsema, 2000).

In the present study, we used fMRI to assess the similarity or divergence of neural effects from spatial precuing (as in many attention studies) and rapid spatial postcuing (as in classic iconic-memory paradigms). It is already known that precued spatial attention can modulate activity in contralateral occipital visual areas (see, e.g., Driver & Frackowiak, 2001; Kastner & Ungerleider, 2000; Serences & Yantis, 2006), but it remains largely unknown whether postcues can do so as well. In fact, there has been very little, if any, neuroimaging work on iconic memory (but see Nobre et al., 2004, for work on pre- and postcues in the context of a working memory paradigm).

We presented brief displays of six visual characters, with one column of three in each hemifield. Only characters in one hemifield had to be judged on each trial. The relevant side was either precued (200 ms prior to display onset) or postcued (200 ms after display offset) with a sound (as in Sperling, 1960). In a behavioral study (Experiment 1), we confirmed that the 200-ms postcue improved performance on the partial-report task, relative to performance with a more delayed (500-ms) postcue; this result is consistent with previous findings on readout from iconic memory. In the fMRI study (Experiment 2), we found that 200-ms precues and postcues produced similar activity changes in lateral occipital cortex contralateral to the cued side, a result indicating that readout from iconic memory may involve modulations of visual cortex similar to those found with precued spatial attention. Also, a comparison with a passive control condition using the same stimuli but no partial-report requirements showed that the 200-ms pre- and postcues activated highly similar networks in fronto-parietal cortex.

## METHOD

### Subjects

Twelve observers (6 males, 6 females; 18 to 32 years old) participated in Experiment 1, and 13 different observers (7 males, 6 females; 19 to 33 years old) participated in Experiment 2. All were healthy, had normal or corrected visual acuity, had no history of neurological or psychiatric illness, and gave written informed consent in accord with local ethics.

### Stimuli, Task, and Setup

All stimuli were generated and presented on a computer using the MATLAB software package Cogent (The Wellcome Trust Centre for Neuroimaging, n.d.). Experiment 1 was performed in a darkened soundproof booth. In Experiment 2, displays were projected onto a rear-projection screen at the back of the scanner bore and viewed by the subjects via a mirror on the head coil. A computer controlled the display in synchronization with fMRI slice acquisition.

**Fig. 1:**
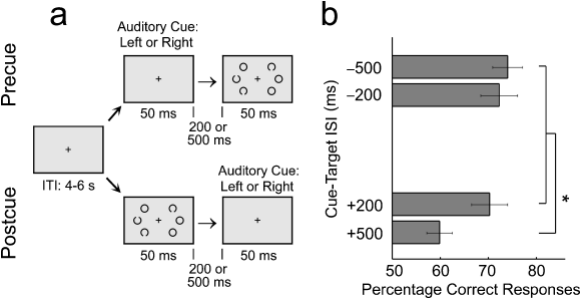
Illustration of the judgment task and behavioral results from Experiment 1. In the two experiments, observers were presented with the same visual displays (a). In the precue condition, an auditory cue preceded a visual display of six Landolt circles (three in each hemifield), and in the postcue condition, the cue followed the visual display. The task was to judge how many circles in the cued hemifield contained a gap (one, two, or three were equiprobable possibilities). The graph (b) shows the percentage of correct responses as a function of cue-stimulus interstimulus interval, or ISI (−500 ms, −200 ms, +200 ms, or +500 ms). Error bars represent the standard errors of the means. Chance performance was 33% correct. An asterisk indicates a significant difference between conditions, *p* < .05. ITI = intertrial interval.

On each trial in both experiments, a brief display (50 ms, presented against a white background) contained three black Landolt circles (each 1.6° in diameter) in each visual hemifield; all six circles were located on an imaginary circle that had a radius of 4° and was centered on the fixation cross (see Fig. 1a). The cue was a sinusoidal tone (1000 Hz, 50-ms duration) presented to the right or left ear (randomly determined for each trial) via pneumatic headphones. The interstimulus intervals (ISIs) at which this cue tone was presented, before or after the visual stimuli, are given in the next paragraphs, in the descriptions of the designs of the two experiments. Some of the visual stimuli (randomly determined) contained a small gap (about 0.4°) at the top or bottom of the circle, and the task was to report, via button press, the number (one, two, or three) of such target stimuli (with the gap) on the cued side. Subjects maintained central fixation, as confirmed with remote optics eye tracking (Experiment 1: ASL 504 R6, at 50 Hz; Experiment 2: ASL 504 LRO, at 60 Hz; both from Advanced Science Laboratories, Bedford, MA). Subjects maintained fixation equally well during all conditions, as determined by a 2 (cued side: left vs. right) × 2 (cue: precue vs. postcue) analysis of variance (ANOVA) of the eye-position data acquired during the fMRI trials (no significant effects or interactions). On average, the eye signal deviated more than 2° from the fixation cross on fewer than 8% of trials, and there were also no significant differences between conditions in eye-position variability, number of blinks, or pupil dilations.

In Experiment 1, we measured accuracy in a 2 (cue: precue vs. postcue) × 2 (ISI: 200 vs. 500 ms) × 2 (cued side: left vs. right) design. The 500-ms postcue was beyond the usual inferred duration of iconic memory (e.g., Sperling, 1960) and thus was expected to lead to impaired performance. In four separate experimental runs, we presented a total of 36 trials per condition (288 trials altogether) in random order, with a 4- to 6-s intertrial interval (randomly determined).

Given the results of the purely behavioral experiment, the fMRI experiment used only the shorter ISIs (i.e., ± 200 ms). These pre- and postcues yielded comparable performance, which ensured that condition was not confounded with task difficulty (such a confound would have undermined interpretation of the fMRI data, but future work might study more delayed postcues). Thus, the fMRI experiment had a 2 (cue: precue vs. postcue) × 2 (cued side: left vs. right) main design. In addition, to account for any differences in brain activation that might simply reflect the different stimulus sequences used for pre- versus postcue trials, rather than the cognitive processes engaged, we presented the equivalent stimuli (i.e., tone before visual array or visual array before tone, with 200-ms ISIs) in corresponding *passive* blocks that did not require any judgment (other than pressing a button after stimulus presentation on each trial, so that motor factors would be equated). Active and passive trials were run separately in randomly ordered miniblocks of 12 trials (randomly determined intertrial interval of 4 to 6 s), with the color of the fixation cross indicating whether the block required judgments (red, active) or no judgments (blue, passive). As in traditional studies of iconic memory, trials with different cue timings (pre- vs. postcue trials) were interleaved. Each subject completed four experimental runs of about 9 min each inside the scanner. A total of 384 trials, 48 per condition, was presented.

### fMRI Procedures and Data Analysis

Functional images were collected on a 3-T Siemens ALLEGRA MR system (Siemens, Erlangen, Germany) with standard head coil, using a multislice gradient-echo echo-planar imaging sequence (32 slices, repetition time = 2,080 ms, 3-mm × 3-mm in-plane resolution, 2-mm slice thickness, 50% spatial gap between adjacent slices). Four runs of 280 volumes each were collected per subject, followed by a T1-weighted anatomical scan.

All functional data were analyzed with SPM2 (Wellcome Trust Centre for Neuroimaging, London, United Kingdom). The first six images of each run were discarded. Images were realigned to the first of the series, movement corrected, adjusted for slice-timing differences, normalized to Montreal Neurological Institute (MNI) anatomical standard space, corrected for global signal-intensity drifts, and spatially smoothed with a three-dimensional 6-mm full-width/half-maximum (FWHM) Gaussian kernel.

For each subject, voxel time series were first regressed onto a composite general linear model containing eight covariates of interest per session (left vs. right side × precue vs. postcue × active vs. passive trials). These covariates represented appropriately placed stick functions convolved with a canonical hemodynamic response function. Low-frequency drifts and short-term temporal autocorrelations were excluded with a high-pass filter (128-s cutoff) and a first-order autoregressive process, respectively. The resulting contrast images were then resmoothed (6-mm FWHM Gaussian kernel) and used for statistical inference in second-level (random-effects) analyses. Minimum-statistic conjunction analyses (Friston, Penny, & Glaser, 2005) were used to test for the presence of any common effects for two independent contrasts (e.g., left minus right for both precues and postcues). Moreover, for hypothesis-driven region-of-interest (ROI) analyses, we extracted SPM parameter estimates from spherical ROIs (6-mm radius), centered at the peaks of the activated clusters. The threshold was set to *p* < .001 and a cluster threshold of *k* > 17 (the expected number of voxels per cluster given the image smoothness and degrees of freedom of the model). Note, however, that lowering the cluster threshold to *k* > 0 did not reveal any effect that is critically absent in the results reported here (e.g., no activation ipsilateral to the cued side in analyses of lateralized activity modulations). Peak voxel coordinates are reported in the MNI space used by SPM2.

## RESULTS

### Experiment 1

We report results collapsed across hemifield, as that factor had no influence on behavior. The visual stimuli in our partial-report task were processed with comparable accuracy for pre- and postcues at the 200-ms ISI; for postcues (but not precues), accuracy was lower at the 500-ms ISI, as expected (see Fig. 1b). The 2 × 2 ANOVA yielded a significant interaction of cue and ISI, *F*(1, 11) = 5.09, *p*_rep_= .92, η^2^= 31.65. Post hoc pair-wise comparisons revealed that accuracy was significantly lower in the 500-ms postcue condition than in the three other conditions (paired *t* tests, all *d* s > 2.4, all *p*_rep_s > .99). In contrast, performance in the 200-ms postcue condition was similar to performance in both precuing conditions, demonstrating the utility of a rapid postcue, as employed in the classic iconic-memory literature. This pattern accords with the findings of many previous partial-report studies on the inferred duration of iconic memory (e.g., Sperling, 1960).

### Experiment 2

Our fMRI experiment included only the −200-ms and +200-ms ISIs, as these led to comparable performance for precues and postcues (the significant increase in task difficulty for 500-ms postcues would have precluded clear interpretations of any differences in brain activity). The 200-ms precue and the 200-ms postcue would classically be associated with selective attention and readout from iconic memory, respectively. Overall, behavioral performance was slightly worse during fMRI scanning (68% correct responses for precues, 63% correct responses for postcues) than in Experiment 1, but was still comparable, as confirmed by a 2 (experiment: 1 vs. 2) × 2 (cue: precue vs. postcue) mixed-design ANOVA that yielded neither main effects nor an interaction, all *F* s(1, 46) < 1.5.

#### Pre- and Postcues: Lateralized Activity Modulations in Occipital Cortex

Given prior neuroimaging findings for precued spatial attention (e.g., Corbetta & Shulman, 2002; Driver & Frackowiak, 2001; Kastner & Ungerleider, 2000), one would expect that the active trials in the 200-ms precue condition would show some increases in occipital activity in the hemisphere contralateral to the cued side, as that occipital hemisphere processes the cued hemifield. The more novel question in this study was whether such a pattern would also be found in the active 200-ms postcue condition, or whether a completely different outcome (possibly no effects within visual cortex) would be found for such readout from iconic memory.

**Fig. 2:**
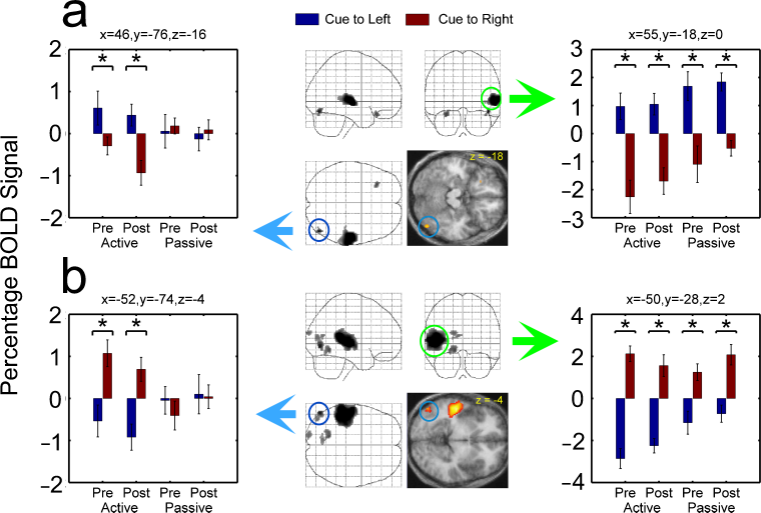
Results for regions showing activity modulations related to cued target side on both active precue and active postcue trials. The brain images in the center of the figure show the areas with stronger activation (a) for active pre- and postcues to the left hemifield than for active pre- and postcues to the right hemifield and (b) for active pre- and postcues to the right hemifield than for active pre- and postcues to the left hemifield. These activations are displayed on a three-dimensional transparent brain template and on transverse slices of the mean structural scan of the subjects (different shades of gray and different warm colors indicate different *p* values for the comparison in question; all *p* s < .001). For each comparison, the area exhibiting peak activation in occipital cortex is circled in blue, and the area exhibiting peak activation in temporal cortex is circled in green. The color-coded arrows point to graphs showing mean blood-oxygenation-level-dependent (BOLD) signal in the circled regions (extracted from 6-mm spherical regions of interest centered in the *x, y*, *z* coordinates given above the graphs) during the active and passive conditions. Asterisks indicate significant (*p* < .05) differences between conditions. Pre = precue trials; Post = postcue trials.

Our fMRI data showed that pre- and postcues elicited very similar modulatory effects on activity in occipital cortex contralateral to the cued hemifield, which contained the to-be-reported stimuli. Figure 2 shows results from two conjunction analyses (see Method) identifying regions with greater activity for left-cued than right-cued active trials, or vice versa, in both the precue and postcue conditions. Two symmetric regions in left and right lateral occipital cortex (middle occipital gyri, circled in blue in Fig. 2) showed higher activity during partial report for contralaterally than for ipsilaterally cued trials. For one of the two comparisons (right minus left), some medial occipital regions in the target-contralateral hemisphere also showed such a response pattern, but this activation was not present symmetrically for the complementary analysis (left minus right). No occipital regions showed significantly lateralized activity modulations specific to either pre- or postcue trials (i.e., we found no significant interaction of cued side and precue/postcue on active trials).

The left panels in Figure 2 show mean blood-oxygenation-level-dependent (BOLD) signals extracted from the symmetric lateral occipital regions circled in blue. Mean BOLD signal intensity was higher during partial reports of contralateral than ipsilateral visual stimuli, for both the 200-ms precue and the 200-ms postcue trials; 2 (cued side: left vs. right) × 2 (cue: precue vs. postcue) ANOVAs on extracted data showed main effects of cued side, all *F* s(1, 12) > 15.5, all *p*_rep_s > .98, all η^2^s > 56.48. The contralateral-ipsilateral difference was comparable for pre- and postcues (i.e., no significant interactions), all *F* s(1, 12) < 0.59. The corresponding passive conditions did not show any effects of the side of the auditory cue on these occipital regions; there were neither main effects nor interactions in corresponding 2 × 2 ANOVAs of the passive conditions, all *F* s(1, 12) < 0.60. This confirms that the activity changes observed in target-contralateral occipital cortex during active pre- and postcue trials did indeed index task-dependent processes, not simply low-level stimulus interactions that could reflect the mere ordering and relative locations of sounds and visual displays.

We also assessed whether the modulations of target-contralateral occipital cortex on active trials might relate to behavioral performance. Pooling over active conditions (i.e., pre- and postcues), we found a positive subject-by-subject correlation between percentage correct and fMRI signal difference (contralateral minus ipsilateral targets) for the right lateral occipital region circled in Figure 2, *r*(11) = .54, *p*_rep_= .91. The corresponding region in left occipital cortex showed only a nonsignificant trend for such a relationship, *r*(11) = .11. Note that similar correlations were observed in separate analyses using only the critical data from active postcue trials, *r*(11) = .51, *p*_rep_= .89, for the right hemisphere and *r*(11) = .17, *p*_rep_= .65 for the left hemisphere.

Unlike the lateral occipital regions, which showed activity modulations contralateral to the cued hemifield exclusively on active trials, regions in auditory temporal cortex (see regions circled in green and the right panels in Fig. 2) displayed lateralized activity increases that were comparable during active and passive trials (i.e., main effects of cued side in corresponding ANOVAs), all *F* s(1, 12) > 18.62, all *p*_rep_s > .99, all η^2^s > 60.81; there were no significant interactions between task and cued side, all *F* s(1, 12) < 2.01. These activity changes thus appear to reflect purely stimulus-driven responses to the monaural sound, which was present in both the active and the passive conditions at one ear or the other. This divergence of cue effects in auditory cortex (not dependent on task) and occipital cortex (found only for the active pre- and postcue trials) again demonstrates that low-level cross-modal effects cannot account for the task-dependent activity modulations in lateral occipital cortex.

#### Control Structures Implicated in Selective Attention and in Readout From Iconic Memory

**Fig. 3:**
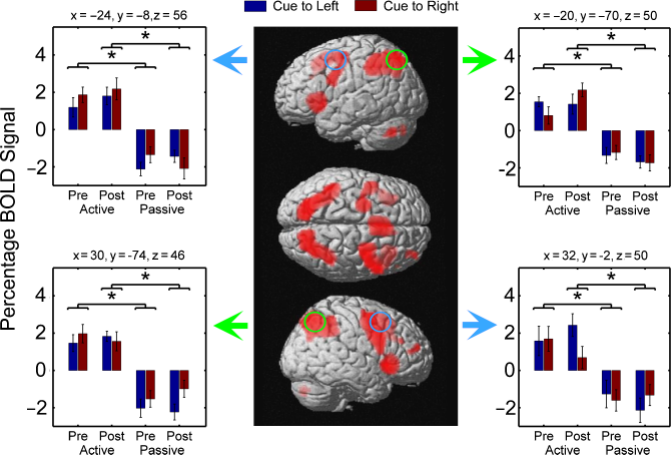
Results for regions activated on both active precue and active postcue trials irrespective of cued side. The brain images in the middle show the activations elicited by both active precues and active postcues (relative to passive precues and passive postcues, respectively), displayed on the standard template brain image employed in SPM2. Different shades of red represent different distances of activations from the cortical surface. These plots show activation of a bilateral network comprising, among other regions, the superior parietal lobule and the human homologue of the frontal eye-fields (circled regions). The arrows point to graphs showing mean blood-oxygenation-level-dependent (BOLD) signal in the regions circled in the same color (BOLD signals were extracted from spherical regions of interest centered in the *x, y, z* coordinates given above the graphs) during the active and passive conditions. Asterisks indicate significant differences in paired *t* tests, *d* > 2.25, *p*_rep_ > .98. Pre = precue trials; Post = postcue trials.

We also assessed our fMRI data to identify any regions involved in both pre- and postcue trials, independently of which side was cued. For the precue conditions, one might expect activation in the distributed fronto-parietal network often associated with control of spatial selective attention (Corbetta & Shulman, 2002; Serences & Yantis, 2006). The new question was whether similar or different structures would be implicated in control of readout from iconic memory (with 200-ms postcues). To answer this question, we first compared active and passive trials (collapsed across target side), testing for conjunctions across the pre- and postcue conditions. This comparison was intended to identify activations present for both active precue and active postcue trials, relative to the passive trials with equivalent sensory input. We found strong activity in a bilateral network of frontal and parietal brain regions (see Fig. 3), including the bilateral superior parietal lobule and the human frontal eye fields. Although this pattern is consistent with numerous reports of such a network during cue-guided attentional processing (Corbetta & Shulman, 2002; Serences & Yantis, 2006), in this study these activity increases were not specific to the precue conditions, but were also present during postcue trials (see the signal plots on both sides of Fig. 3).

**Fig. 4:**
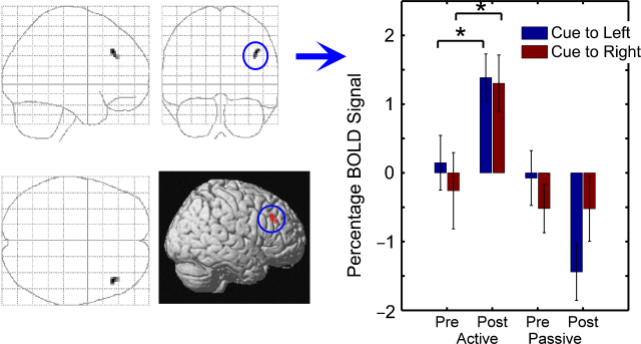
Results for the region with larger task-dependent activity for active postcues than precues. The brain panels show the activations found for postcues relative to precues, irrespective of side, on active trials. These activations, in right middle frontal gyrus, are displayed on a three-dimensional transparent brain template and on the standard template brain image employed in SPM2. The graph shows the mean blood-oxygenation-level-dependent (BOLD) signal extracted from a spherical region of interest (indicated by the blue circle) centered in this region. Results are shown as a function of type of cue (precue vs. postcue), side of cue (left vs. right), and type of trial (active vs. passive). Asterisks indicate significant (*p* < .05) differences between conditions. Pre = precue trials; Post = postcue trials.

None of these nonlateralized activations in frontal and parietal regions, found for the comparison of active versus passive trials, were larger during precue trials than during postcue trials. However, we found some symmetrical occipital regions (peak at *x* = −2, *y* = −84, *z* = −6), in more medial occipital structures such as the cuneus, that showed higher activity during precue trials than during postcue trials. Inspection of the BOLD signals from these regions revealed a trend for stronger activation for contralateral than ipsilateral stimuli, but this laterality did not reach statistical significance and hence was not detected in the analyses of lateralized activity modulations described earlier (see Fig. 2). For the inverse comparison (postcues minus precues in the active condition), we found a single region in right middle frontal gyrus (peak at *x* = 42, *y* = 30, *z* = 34) that was more active during postcuing than during precuing, *F*(1, 12) = 11.74, *p*_rep_= .98, η^2^= 49.45, irrespective of which side had been cued, *F*(1, 12) = 0.078 (see Fig. 4). Again, this effect was specific to the active trials, as no such difference was found for the passive conditions.

## DISCUSSION

Most cognitive psychology textbooks present selective attention and iconic memory as separate topics. But these topics have often been studied with comparable partial-report paradigms, which may differ only in whether cues are administered before or shortly after the visual displays (see also Bundesen, 1990; Shih & Sperling, 2002). Here we have shown that 200-ms precues and postcues can lead to comparable partial-report performance for identical displays (a more delayed postcue caused a decrement in Experiment 1) and can elicit overlapping activity modulations in lateral occipital cortex. We also found that similar bilateral networks of frontal and parietal regions were activated in pre- and postcue conditions. These effects provide neural confirmation for functional similarities of partial report in the context of pre- and postcues, as traditionally used in the study of selective spatial attention and iconic memory, respectively.

Our findings imply that pre- and postcues, although differing logically, may draw on similar neural processes to aid performance. This surprising finding might reflect the prolonged and highly recursive nature of cortical processing. Visual input entering visual cortex is not merely conveyed to higher-level brain regions in a strictly serial fashion, but rather is processed in multiple parallel feedback loops (Felleman & Van Essen, 1991). As a consequence, neural activity related to a particular visual stimulus can persist over several hundred milliseconds (Bullier, 2001; Lamme & Roelfsema, 2000), lasting substantially beyond stimulus offset in the case of brief displays. The lateralized activity modulations in lateral occipital cortex found here for both 200-ms precues and 200-ms postcues may thus represent similar processes devoted to selectively enhancing such persistent neuronal activity. Our results imply that at least some of the “iconic memory” traces classically assumed to underlie postcue partial-report advantages (Neisser, 1967; Sperling, 1960) may relate to ongoing cortical processing in higher visual areas, such as lateral occipital cortex (Grill-Spector, Kourtzi, & Kanwisher, 2001; Malach et al., 1995), as found here. The time course and decay of iconic memory might reflect temporal properties of recursive computations in such higher visual areas, where percept-related activity has been shown to persist after stimulus offset (Ferber, Humphrey, & Vilis, 2005; Mukamel, Harel, Hendler, & Malach, 2004).

Another similarity in the neural effects of 200-ms precues and 200-ms postcues in this study concerned nonlateralized activity changes in regions well beyond occipital visual cortex. In the active condition, a bilateral fronto-parietal network was activated during both pre- and postcue trials, with only minor differences (see the next paragraph) between these types of trials. In the attention literature, it is often argued that activity in such brain structures may reflect top-down control processes that modulate activity in visual cortex to enhance perception of target stimuli relative to distractors (Desimone & Duncan, 1995; Duncan, Humphreys, & Ward, 1997; Kastner & Ungerleider, 2000; Ruff et al., 2006; Serences & Yantis, 2006). The largely identical neural activity in fronto-parietal areas found here for pre- and postcue partial report implies that readout from iconic memory may involve control processes similar to those underlying selective attention.

In the present study, only one brain area, the right middle frontal gyrus, was found to be more active during post- than precue trials. This greater activation might reflect control processes specific to readout from iconic memory, or to accessing internal representations more generally (see also Nobre et al., 2004). Activation of a comparable section of the right middle frontal gyrus was found in a previous study when participants had to select one of several spatial locations maintained in working memory (Rowe, Toni, Josephs, Frackowiak, & Passingham, 2000). Although access to internal representations might conceivably be more important for postcued than precued partial report, we emphasize again that the right middle frontal gyrus region was the only area to exhibit greater activation in the postcue than the precue condition, whereas we found many activations common to these two conditions in other fronto-parietal regions.

The only regions showing higher activity during pre- than postcue trials were in more medial occipital cortex; these findings possibly indicate alerting or uniquely attentional effects. But these regions showed only a trend for contralaterality. The stronger, significantly contralateral effects found in more lateral occipital structures (see Fig. 2) may have arisen because task-related modulation of visual responses is typically stronger for higher visual areas (Kastner & Ungerleider, 2000) or because our task involved shape perception, for which lateral occipital cortex may be specialized (Grill-Spector et al., 2001; Malach et al., 1995).

In conclusion, our study provides fMRI evidence that very similar processes may underlie partial report in the context of pre- and postcues, as traditionally used to study selective attention and iconic memory, respectively. We found similar modulations of lateral occipital activity, as well as common activity in a bilateral fronto-parietal network, during precued and postcued partial report. These data may provide evidence against proposals that postcued reports of visual information rely primarily on postperceptual categorical processing (e.g., Coltheart, 1980), beyond the visual system. Our findings seem more consistent with the idea that the iconic memory measured by rapidly postcued partial report relates to enduring stimulus-related activity in occipital cortex, which may be read out by control processes in fronto-parietal regions similar to those implicated in spatial selective attention.
